# Nonlinear Steering Wheel Angle Control Using Self-Aligning Torque with Torque and Angle Sensors for Electrical Power Steering of Lateral Control System in Autonomous Vehicles

**DOI:** 10.3390/s18124384

**Published:** 2018-12-11

**Authors:** Wonhee Kim, Chang Mook Kang, Young-Seop Son, Chung Choo Chung

**Affiliations:** 1 School of Energy Systems Engineering, Chung-Ang University, Seoul 06974, Korea; whkim79@cau.ac.kr; 2 Department of Electrical Engineering, Hanyang University, Seoul 04764, Korea; kcm0728@hanyang.ac.kr; 3 Automotive Electronics R&D Center, CAMMSYS Corporation, Incheon 22013, Korea; ysson@cammsys.net; 4 Division of Electrical and Biomedical Engineering, Hanyang University, Seoul 04764, Korea

**Keywords:** electric power steering, angle control, self-aligning torque, backstepping control, augmented observer

## Abstract

The development of sensor technology enabled the use of composite sensors to measure the torque and angle of steering wheels at gradually decreasing costs while maintaining the required safety. The electric power steering (EPS) is vital to the safety of the car, therefore it is not worth sacrificing safety to save cost and the SWA control with angle sensor gradually becomes the mainstream. Existing methods to control steering wheel angle (SWA) for EPS consider the self-aligning torque as a disturbance that should be rejected. However, this torque is useful to return the SWA from an outward to the center position. Hence, we propose a nonlinear control of SWA using the self-aligning torque for EPS in the lateral control system of autonomous vehicles. The proposed method consists of a high-gain disturbance observer and a backstepping controller, where the former aims to estimate the self-aligning torque, and an auxiliary state variable prevents using the derivative of the measured signal. The nonlinear controller is designed via backstepping to bound the SWA tracking error. The self-aligning torque provides damping that can improve the controller tracking when following the same direction of the input torque on the steering wheel control. In this case, the control input can be reduced by the damping effect of the self-aligning torque. The performance of the proposed method is validated through EPS hardware-in-the-loop simulation.

## 1. Introduction

Enhancing the safety and comfort to autonomous vehicle drivers is an important concern in the automotive industry and can be achieved by proper motion control. Vehicle motion control of autonomous vehicles can be classified into lateral and longitudinal methods. Longitudinal control aims to maintain the distance necessary from the controlled vehicle to the preceding one to prevent collision [1,2]. This control implements approaches such as adaptive cruise control that ensures a preset velocity and a safe distance among vehicles, being the most widely used advanced driver-assistance system in the automotive industry nowadays. On the other hand, lateral control aims to either maintain the vehicle within the intended lane or track the desired lateral position [3,4,5,6,7]. Various lateral control methods are available. For instance, a linear control method has been developed using lead-lag control [8]. Four lane keeping control methods have been compared in [9]. In [10], potential-field-based lane keeping control was developed to guarantee stability and robustness, whereas multi-rate control was designed to improve the lateral control performance in [11]. Moreover, an adaptive PID neural-network controller has been proposed for lateral tracking control of intelligent vehicles [12], and a hierarchical lateral control scheme for autonomous vehicles was developed considering uneven time delays induced caused by vision sensors [13]. Recently, an integrated longitudinal and lateral networked control system was proposed for vehicle platooning [14]. In all these lateral control methods, the desired steering wheel angle (SWA) is regulated using the vehicle lateral dynamics.

Generally, the angular velocity estimation method was widely used to save cost because the price of angle sensor is higher. Then the SWA was estimated using integration of the angular velocity. Currently, electric power steering (EPS) is increasingly replacing the conventional hydraulic power steering, which is still widely used for heavy vehicles [15], given its superior performance in several aspects including safety, cost, energy efficiency, environmental protection, and assembly process [4,15,16,17]. The development of sensor technology enabled the use of composite sensors to measure the torque and angle of steering wheels at gradually decreasing costs while maintaining the required safety. Thus, the torque and angle sensor (TAS) is installed to measure the SWA and torque in the EPS. Furthermore, the resolver is used to measure the motor angle in the EPS. The EPS is vital to the safety of the car, therefore it is not worth sacrificing safety to save cost and the SWA control with angle sensor gradually becomes the mainstream [17].

When the driver manually handles the steering wheel, the main role of the EPS system is torque control of the motor to generate the assistant torque. The assistant torque and driver torque are then combined to make the tires turn. Thus, various torque control methods for EPS have been proposed [18,19,20,21]. On the other hand, in the autonomous vehicle, the main role of the EPS system is to make the SWA track the desired SWA derived by the lateral control method. In previous lateral control methods, the SWA has been considered as the input for the controller, and either a DC motor instead of a EPS system or a modified EPS system allowing SWA control has been employed. Recently, SWA control with neither an additional DC motor nor EPS modifications has been proposed [22,23,24]. In [22], the EPS system function, external disturbances, and input gain uncertainty are considered as lumped disturbance. In [23], an intelligent sliding-mode control implemented on a digital signal processor performs the EPS position control. In [24], a systematic approach to identify the steering behavior and design a nonlinear controller by utilizing experimental data is proposed.

Although previous methods improve SWA tracking, a relatively large SWA tracking error can be caused by aspects such as hysteresis and friction, especially when the direction of the SWA changes. In addition, external lateral force disturbance is cancelled to further improve SWA tracking, thus removing the effect of the self-aligning torque. In fact, this torque is the main component of external lateral force disturbance and occurs between the contact patch of the tire tread and the road surface. When the tires are controlled to turn outward, the self-aligning torque is considered as a disturbance to be overcome in these methods. However, the self-aligning torque could be used in controllers for the steering wheel to return to the center position after turning in corners, becoming into a useful variable of external lateral force for SWA control. Moreover, the self-aligning torque can be especially useful to return to the center position of the SWA after its direction changes [25,26]. Consequently, previous methods have the drawback of neglecting this useful part in the lumped disturbance, ignoring the effect of the self-aligning torque and possibly generating an increase of the control input to unnecessarily cancel its effect in some situations.

In this paper, we propose a nonlinear SWA control considering the self-aligning torque for EPS in lateral control systems of autonomous vehicles. The main motivation is the use of the self-aligning torque to improve the SWA tracking performance. The proposed method consists of a high-gain disturbance observer (HGDOB) and a backstepping controller, where the former aims to estimate the self-aligning torque with an auxiliary state variable to prevent the use of the measured signal derivative. Then, a nonlinear controller is designed via backstepping to guarantee the boundedness of the SWA tracking error. The self-aligning torque aims to introduce a damping effect to improve the tracking performance of the controller when the input torque on the steering wheel has the same direction of the self-aligning torque, thus reducing the control input. The main contributions of this study are summarized as follows:

- Nonlinear damping using the self-aligning torque improves the SWA tracking performance and reduces the control input in the backstepping controller.
- An auxiliary state variable prevents using the measured signal derivative in the HGDOB.
- The performance of the proposed method is validated via EPS hardware-in-the-loop (HILS) simulation.

## 2. EPS System Model

We consider a column-type EPS system consisting of a steering wheel, intermediate shaft, motor, torque and angle sensor, reduction gear, and rack/pinion structure, as detailed in [11]. The driver torque and SWA are measured by the torque and angle sensor that sends the measured values to the electronic control unit. The resolver in the motor measures the motor angle. In autonomous vehicles, the primary role of the EPS system is to make SWA $\theta_{h}$ track desired SWA $\theta_{h_{d}}$, derived by the lateral control method. Considering Newton’s laws of motion, the EPS model can be represented in the state-space form [20] as

(1) $$\begin{array}{cc} {\dot{\theta }}_{h} & =\omega_{h}\\ {\dot{\omega }}_{h} & =\frac{1}{J_{c}}\left(-K_{c}\theta_{h}-B_{c}\omega_{h}+\frac{K_{c}}{N}\theta_{m}+T_{d}\right)\\ {\dot{\theta }}_{m} & =\omega_{m}\\ {\dot{\omega }}_{m} & =\frac{1}{J_{eq}}\left(\frac{K_{c}}{N}\theta_{h}-K_{N}\theta_{m}-B_{eq}\omega_{m}+T-\frac{R_{p}}{N}T_{s}\right) \end{array}$$

where $J_{eq}=J_{m}+\frac{R_{p}^{2}}{N^{2}}M_{r}$, $K_{N}=\frac{K_{c}+K_{r}R_{p}^{2}}{N^{2}}$ and $B_{eq}=B_{m}+\frac{R_{p}^{2}}{N^{2}}B_{r}$. In this study, we assume that driver torque $T_{d}$ can be neglected given the autonomy of the vehicle. The expression in (1) can be compactly rewritten as

(2) $$\begin{array}{c} \begin{array}{c} {\dot{x}}_{1}=x_{2}\\ {\dot{x}}_{2}=a_{21}x_{1}+a_{22}x_{2}+a_{23}x_{3}\\ {\dot{x}}_{3}=x_{4}\\ {\dot{x}}_{4}=a_{41}x_{1}+a_{43}x_{3}+a_{44}x_{4}+b_{4}u-d \end{array} \end{array}$$

where $x={\left[x_{1},\;x_{2},\;x_{3},\;x_{4}\right]}^{T}={\left[\theta_{h},\;\omega_{h},\;\theta_{m},\;\omega_{m}\right]}^{T}$, u=T, $a_{21}=-\frac{K_{c}}{J_{c}}$, $a_{22}=-\frac{B_{c}}{J_{c}}$, $a_{23}=\frac{K_{c}}{J_{c}N}$, $a_{41}=\frac{K_{c}}{J_{eq}N}$, $a_{43}=-\frac{K_{N}}{J_{eq}}$, $a_{44}=-\frac{B_{eq}}{J_{eq}}$, $b_{4}=\frac{1}{J_{eq}}$, and $d=\frac{R_{p}}{J_{eq}N}T_{r}$. Term $-\frac{R_{p}}{N}T_{s}$ is regarded as the unknown disturbance *d*.

## 3. High Gain Disturbance Observer

From (2), the dynamics of disturbance *d* can be expressed as

(3) $$d=-{\dot{x}}_{4}+a_{41}x_{1}+a_{43}x_{3}+a_{44}x_{4}+b_{4}u.$$

Next, we denote the estimate of the disturbance as $\hat{d}$, whose estimation error is defined as

(4) $$\tilde{d}=d-\hat{d}.$$

The dynamics of $\hat{d}$ are given by

(5) $$\dot{\hat{d}}=\frac{1}{\varepsilon }\left(-{\dot{x}}_{4}+a_{41}x_{1}+a_{43}x_{3}+a_{44}x_{4}+b_{4}u-\hat{d}\right)$$

where $\frac{1}{\varepsilon }$ is the observer gain. To suppress the bounded derivative of the disturbance, a high gains, i.e., low value of ε is required. However, as measurement noise appears in the sensors, and the dynamics of $\hat{d}$ in (5) use the derivative of the state, then HGDOB would amplify the noise given its high gain, impeding the practical implementation of the observer. To avoid this problem, we use auxiliary state variable ξ defined as

(6) $$\xi =\hat{d}-\frac{x_{4}}{\varepsilon }.$$

whose derivative with respect to time is given by

(7) $$\dot{\xi }=\dot{\hat{d}}-\frac{{\dot{x}}_{4}}{\varepsilon }.$$

Substituting (7) into the disturbance estimation error in (4) with respect to time results in the auxiliary state variable dynamics:

(8) $$\dot{\xi }=-\frac{1}{\varepsilon }\left(\xi +\frac{x_{4}}{\varepsilon }\right)+\frac{1}{\varepsilon }\left(a_{41}x_{1}+a_{43}x_{3}+a_{44}x_{4}+b_{4}u\right).$$

From (3), (4), (6), and (8), the following dynamics of the disturbance estimation error is obtained as

(9) $$\dot{\tilde{d}}=-\frac{1}{\varepsilon }\tilde{d}+\dot{d}.$$

Generally, prior information about the derivative of the disturbances is unknown but at least locally bounded [27]. Therefore, $|\tilde{d}|\leq e^{-\frac{1}{\varepsilon }t}\cdot \left|\tilde{d}\left(0\right)\right|+\varepsilon \rho \left(t\right)$ for envelope function ρ(t) such that $\rho \left(t\right)\geq |\dot{d}|$, ∀t≥0, from which the upper bound of $|\tilde{d}\left(\infty \right)|$ becomes smaller as ε decreases. Note that the proposed disturbance observer in (8) with the auxiliary state variable defined in (6) is independent from derivative ${\dot{x}}_{4}$. Consequently, if (6) and (8) are used instead of (5) to estimate the disturbance, the amplification of measurement noise caused by the high gain is negligible in practice.

## 4. Backstepping Control Using Self-Aligning Torque

### 4.1. Backstepping Controller

We define $x_{1_{d}}=\theta_{h_{d}}$ and the desired SWA, and hence, the tracking error can be expressed as

(10) $$e_{i}=x_{i}-x_{i_{d}}$$

for i∈[1,4]. The dynamics of $e_{1}$ is given by

(11) $${\dot{e}}_{1}=x_{2}-{\dot{x}}_{1_{d}}.$$

Then, we define $V_{1}$ as

(12) $$V_{1}=\frac{1}{2}e_{1}^{2}$$

whose derivative is given by

(13) $$\begin{array}{c} {\dot{V}}_{1}=e_{1}{\dot{e}}_{1}\\ \;=e_{1}\left(x_{2}-{\dot{x}}_{1_{d}}\right)\\ \;=e_{1}\left(x_{2_{d}}+e_{2}-{\dot{x}}_{1_{d}}\right). \end{array}$$

We also design

(14) $$x_{2_{d}}=-k_{1}e_{1}+{\dot{x}}_{1_{d}}$$

where $k_{1}$ is a positive constant, to express ${\dot{V}}_{1}$ as

(15) $${\dot{V}}_{1}=-k_{1}e_{1}^{2}+e_{1}e_{2}.$$

In addition, we define function $V_{2}$ as

(16) $$V_{2}=\frac{1}{2}e_{1}^{2}+\frac{1}{2}e_{2}^{2}$$

whose derivative is given by

(17) $$\begin{array}{cc} {\dot{V}}_{2} & =-k_{1}e_{1}^{2}+e_{1}e_{2}+e_{2}{\dot{e}}_{2}\\ & =-k_{1}e_{1}^{2}+e_{1}e_{2}+e_{2}\left(a_{21}x_{1}+a_{22}x_{2}+a_{23}x_{3}-{\dot{x}}_{2_{d}}\right)\\ & =-k_{1}e_{1}^{2}+e_{1}e_{2}+e_{2}\left(a_{21}x_{1}+a_{22}x_{2}+a_{23}x_{3_{d}}+a_{23}e_{3}-{\dot{x}}_{2_{d}}\right). \end{array}$$

Similarly, we design

(18) $$x_{3_{d}}=\frac{1}{a_{23}}\left(-k_{2}e_{2}-a_{21}x_{1}-a_{22}x_{2}+{\dot{x}}_{2_{d}}-e_{1}\right)$$

where $k_{2}$ is a positive constant, to express ${\dot{V}}_{2}$ as

(19) $${\dot{V}}_{2}=-k_{1}e_{1}^{2}-k_{2}e_{2}^{2}+a_{23}e_{2}e_{3}.$$

Next, we define function $V_{3}$ as

(20) $$V_{3}=\frac{1}{2}e_{1}^{2}+\frac{1}{2}e_{2}^{2}+\frac{1}{2}e_{3}^{2}$$

whose derivative is given by

(21) $$\begin{array}{c} {\dot{V}}_{3}=-k_{1}e_{1}^{2}-k_{2}e_{2}^{2}+a_{23}e_{2}e_{3}+e_{3}{\dot{e}}_{3}\\ \;=-k_{1}e_{1}^{2}-k_{2}e_{2}^{2}+a_{23}e_{2}e_{3}+e_{3}\left(x_{4}-{\dot{x}}_{3_{d}}\right)\\ \;=-k_{1}e_{1}^{2}-k_{2}e_{2}^{2}+a_{23}e_{2}e_{3}+e_{3}\left(x_{4_{d}}+e_{4}-{\dot{x}}_{3_{d}}\right). \end{array}$$

and define

(22) $$x_{4_{d}}=-k_{3}e_{3}+{\dot{x}}_{3_{d}}-a_{23}e_{2}$$

where $k_{3}$ is a positive constant, to obtain

(23) $${\dot{V}}_{3}=-k_{1}e_{1}^{2}-k_{2}e_{2}^{2}-k_{3}e_{3}^{2}+e_{3}e_{4}.$$

Finally, we define $V_{4}$ as

(24) $$V_{4}=\frac{1}{2}e_{1}^{2}+\frac{1}{2}e_{2}^{2}+\frac{1}{2}e_{3}^{2}+\frac{1}{2}e_{4}^{2}$$

whose derivative is given by

(25) $$\begin{array}{c} {\dot{V}}_{4}=-k_{1}e_{1}^{2}-k_{2}e_{2}^{2}-k_{3}e_{3}^{2}+e_{3}e_{4}+e_{4}{\dot{e}}_{4}\\ \;=-k_{1}e_{1}^{2}-k_{2}e_{2}^{2}-k_{3}e_{3}^{2}+e_{3}e_{4}+e_{4}\left(a_{41}x_{1}+a_{43}x_{3}+a_{44}x_{4}+b_{4}u-d-{\dot{x}}_{4_{d}}\right). \end{array}$$

with

(26) $$u=\frac{1}{b_{4}}\left(-k_{4}e_{4}-a_{41}x_{1}-a_{43}x_{3}-a_{44}x_{4}+\left(1-n_{d}\right)\hat{d}-e_{3}\right)$$

where $k_{4}$ is a positive constant and $n_{d}$ is the damping gain of the self-aligning torque. Therefore, the self-aligning torque is canceled if $n_{d}=0$ by control input *u* (26), and considered for control if $n_{d}=1$ Thus, ${\dot{V}}_{4}$ becomes

(27) $${\dot{V}}_{4}=-k_{1}e_{1}^{2}-k_{2}e_{2}^{2}-k_{3}e_{3}^{2}-k_{4}e_{4}^{2}-e_{4}\left(d-n_{d}\hat{d}\right).$$

Consequently, we can express the error dynamics as

(28) $$\dot{e}=A_{e}e+B_{d}\delta$$

where

$$A_{e}=\left[\begin{array}{cccc} -k_{1} & 1 & 0 & 0\\ -1 & -k_{2} & 1 & 0\\ 0 & -1 & -k_{3} & 1\\ 0 & 0 & -1 & -k_{4} \end{array}\right],B_{e}=\left[\begin{array}{c} 0\\ 0\\ 0\\ 1 \end{array}\right]$$

and $\delta =d-\left(1-n_{d}\right)\hat{d}$. The positive control gains make $A_{e}$ become Hurwitz, and we assume that the self-aligning torque, *d*, is bounded. The estimated self-aligning torque, $\hat{d}$ is bounded by the HGDOB. Thus, the upper boundedness $\delta_{\max}$ of |δ| exists such that $\delta_{\max}=\sup_{t}\left|\delta \left(t\right)\right|$. Let us consider the stability of the dynamics of *e* in (28). We define the Lyapunov function $V_{e}$ as

(29) $$V_{e}=e^{T}P_{e}e$$

where $P_{e}$ is positive definite such that $A_{e}^{T}P_{e}+P_{e}A_{e}=-I$. The derivative of $V_{e}$ with respect to time is

(30) $$\begin{array}{c} {\dot{V}}_{e}=e^{T}A_{e}^{T}P_{e}+P_{e}A_{e}e+2e^{T}P_{e}B_{d}\delta \\ \;\leq -{∥e∥}_{2}^{2}+2\delta_{\max}∥P_{e}{∥}_{2}{∥e∥}_{2}\\ \;=-{\left(1-\eta \right)∥e∥}_{2}^{2}-\eta {∥e∥}_{2}^{2}\\ \;+2\delta_{\max}\lambda_{\max}\left(P_{e}\right){∥e∥}_{2},\;\;0<\eta <1 \end{array}$$

where 0<η<1. Consequently,

(31) $${\dot{V}}_{e}\leq -{\left(1-\eta \right)∥e∥}_{2}^{2},\;\;\forall \;{∥e∥}_{2}\geq \frac{2\lambda_{\max}\left(P_{e}\right)\delta_{\max}}{\eta }$$

showing that *e* is globally ultimately bounded.

### 4.2. SWA Control Using Self-Aligning Torque

**Figure 1:**
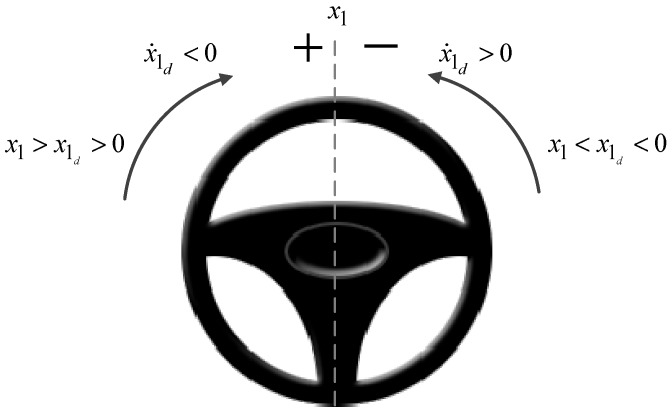
Steering wheel regions for helpful self-aligning torque.

As mentioned above, when the steering wheel is turned and released during cornering, it returns to the center position by the self-aligning torque exerted on the tires by the road. Generally, this torque is considered as a disturbance to SWA control in autonomous vehicles, and previous control methods were designed to reject it. However, the self-aligning torque can be used to return the steering wheel to the center position by itself in autonomous vehicles using its damping effect. Therefore, we propose using the self-aligning torque to improve the performance of SWA control. Figure 1 shows the convenient regions for use of the self-aligning torque, where the left and right sides are denoted with positive and negative signs, respectively. The self-aligning torque is useful for SWA control when either $0<x_{1_{d}}<x_{1}$, ${\dot{x}}_{1_{d}}<0$ or $x_{1}<x_{1_{d}}<0$, ${\dot{x}}_{1_{d}}>0$.

**Figure 2:**
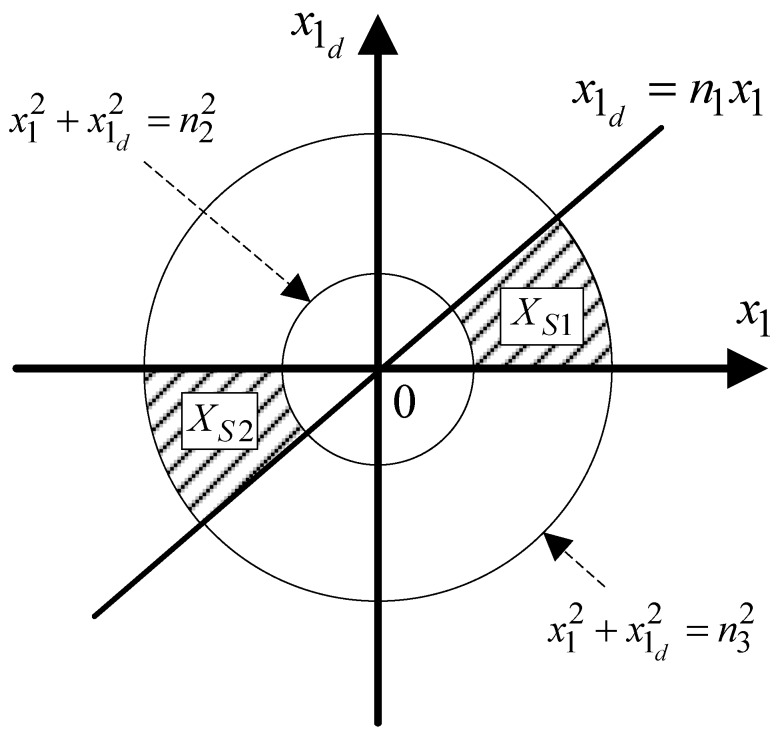
$X_{s1}$ and $X_{s2}$ for $n_{d}$.

Thus, we design damping gain $n_{d}$ of the self-aligning torque as

(32) $$n_{d}=\left\{\begin{array}{cc} 1 & \left(x_{1},x_{1}^{d}\right)\in X_{S1}\;,{\dot{x}}_{1_{d}},\;\mathrm{and}\;V_{x}\in \left({\underline{V}}_{x},{\bar{V}}_{x}\right)<0\\ 1 & \left(x_{1},x_{1}^{d}\right)\in X_{S2},\;{\dot{x}}_{1_{d}},\;\mathrm{and}\;V_{x}\in \left({\underline{V}}_{x},{\bar{V}}_{x}\right)>0\\ 0 & \mathrm{else} \end{array}\right.$$

where regions $X_{s1}$ and $X_{s2}$ are depicted in Figure 2 with $0<n_{1}<1$ and $0<n_{2}<n_{3}$ being the designed factors for $n_{d}$. The self-aligning torque is very low and high at low and high longitudinal velocity, respectively. In these cases, the self-aligning torque is not suitable for use in the controller, as it may deteriorate performance or cause oscillations. However, the self-aligning torque can be suitable for $V_{x}\in \left({\underline{V}}_{x},{\bar{V}}_{x}\right)$ where ${\underline{V}}_{x}$ and ${\bar{V}}_{x}$ indicate the values that satisfy the designed factors. Note that the self-aligning torque increases with both vehicle speed and SWA. Thus, a large self-aligning torque caused by high vehicle speeds and large SWA may cause excessive overshoot and oscillation. Thus, $n_{3}$ is chosen to prevent excessive overshoot and subsequent oscillation.

**Figure 3:**
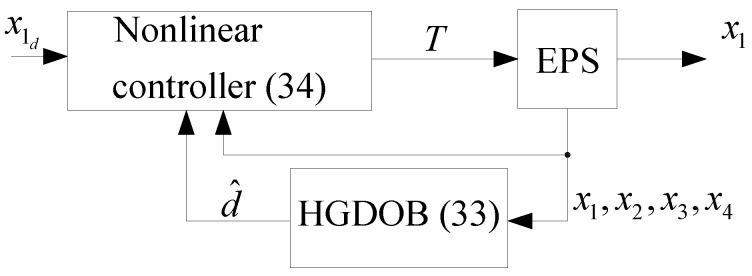
Block diagram of the proposed method.

The proposed controller is expressed as

(33) $$\begin{array}{c} \dot{\xi }=\dot{\hat{d}}-\frac{{\dot{x}}_{4}}{\varepsilon }\\ \hat{d}=\xi +\frac{x_{4}}{\varepsilon } \end{array}$$

(34) $$\begin{array}{c} x_{2_{d}}=-k_{1}e_{1}+{\dot{x}}_{1_{d}}\\ x_{3_{d}}=\frac{1}{a_{23}}\left(-k_{2}e_{2}-a_{21}x_{1}-a_{22}x_{2}+{\dot{x}}_{2_{d}}-e_{1}\right)\\ x_{4_{d}}=-k_{3}e_{3}+{\dot{x}}_{3_{d}}-e_{2}\\ \;u=\frac{1}{b_{4}}\left(-k_{4}e_{4}-a_{41}x_{1}-a_{43}x_{3}-a_{44}x_{4}-b_{4}u+n_{d}\hat{d}-e_{3}\right)\\ \;n_{d}=\left\{\begin{array}{cc} 1 & \left(x_{1},x_{1}^{d}\right)\in X_{S1}\;\mathrm{and}\;{\dot{x}}_{1_{d}}<0\\ 1 & \left(x_{1},x_{1}^{d}\right)\in X_{S2}\;\mathrm{and}\;{\dot{x}}_{1_{d}}>0\\ 0 & \mathrm{else} \end{array}\right. \end{array}$$

and its block diagram is shown in Figure 3. The HGDOB in (33) estimates the self-aligning torque, whereas the backstepping controller in (34) generates the control input using the estimated self-aligning torque and full state feedback.

## 5. Simulations and Experiments

Simulations and experiments were performed to evaluate the performance of the proposed method. In the simulations and experiments, an EPS system (Mando Co., Seoul, Korea) was used. For confidentiality, we do not report the values of the EPS parameters. In [11], it was observed that the absolute value of the maximum steering wheel angle was less than 3 rad (17.2 deg.) and that the dominant frequency was less than 0.05 Hz in the high speed circuit of the Korea Automobile Testing & Research Institute (KATRI) that was designed based on the standard of the highway. Thus, we set the desired SWA as $x_{1_{d}}=0.3\sin\left(0.05\times 2\pi t\right)$ rad for the simulations and experiments.

### 5.1. Simulation Results

**Figure 4:**
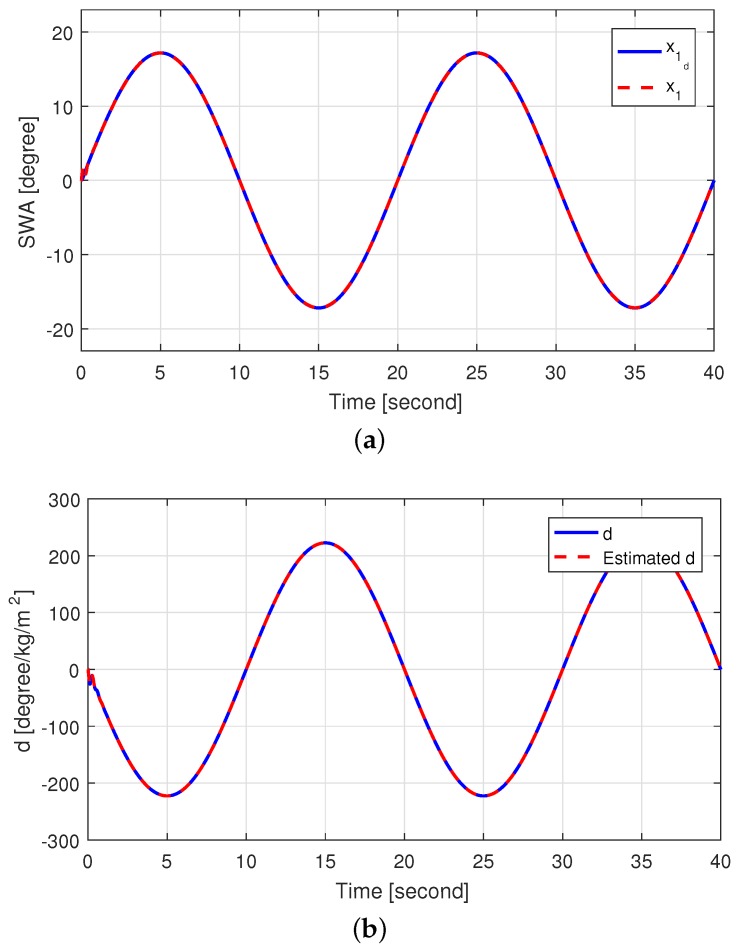
Simulation results (**a**) SWA tracking performance and (**b**) self-aligning torque estimation performance.

In order to evaluate the steering wheel angle tracking and estimation performance, the proposed method was tested via MATLAB/Simulink. The SWA tracking performance and the self-aligning torque estimation performance are shown in Figure 4. It can be seen that the SWA of the EPS matched the desired SWA well even though the self-aligning torque existed. We see that the estimated self-aligning torque tracked the actual self-aligning torque quite well.

### 5.2. Experimental Results

**Figure 5:**
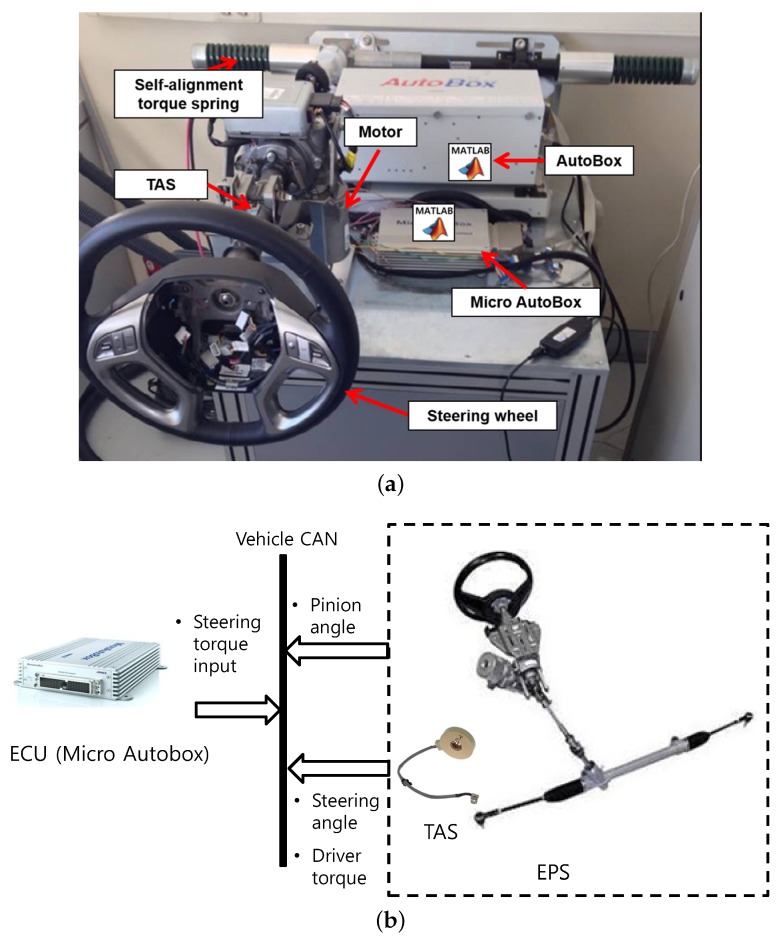
EPS HILS system for experimental verification of SWA controller (CAN, controller area network; ECU, electronic control unit; TAS, torque and angle sensor): (**a**) Photo of the EPS HILS system; (**b**) Block diagram of the EPS HILS system.

We conducted experiments to evaluate the performance of the proposed control method using the EPS hardware-in-the-loop simulation system shown in Figure 5. The system consisted of an EPS system (Mando Co., Seoul, Korea), a spring system, and the AutoBox and MicroAutoBox systems from dSPACE Inc. (Wixom, MI, USA). We mounted a spring on the system to emulate the self-aligning torque. The TAS was used to measure these parameters from the steering wheel, and the motor angle was measured using the embedded resolver. The control algorithm in (33) and (34) was implemented in the MicroAutoBox at a sampling rate of 100 Hz.

To evaluate the damping effect of the self-aligning torque, we tested two cases applying the same control gains:

- **Case 1:** Control without using the self-aligning torque, i.e., $n_{d}=0$
- **Case 2:** Control using the self-aligning torque, i.e., applying (32).

**Figure 6:**
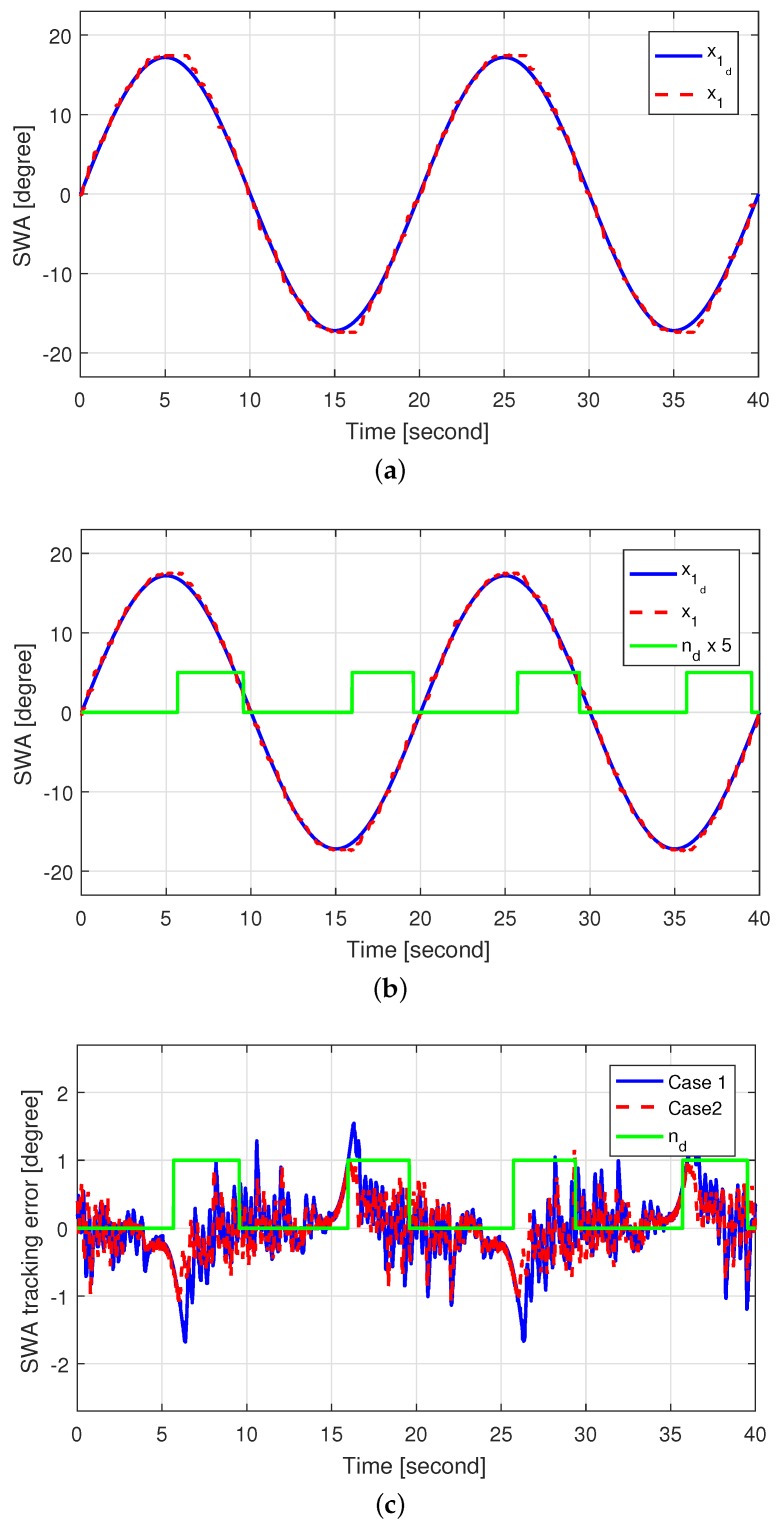
SWA tracking performance: (**a**) SWA tracking performance in case 1; (**b**) SWA tracking performance in case 2; and (**c**) SWA tracking error in cases 1 and 2.

In case 1, the self-aligning torque was always rejected by the controller, whereas in case 2, the self-aligning torque was used for the SWA to return to its center position. After reaching zero angular velocity (i.e., when the SWA changed direction), the SWA was controlled to return to the center position to verify the contribution of the self-aligning torque. The SWA tracking performance for both cases is shown in Figure 6. A relatively large SWA tracking error appeared by aspects such as hysteresis and friction in case 1 near the zero angular velocity, whereas the self-aligning torque was used for damping when $n_{d}=1$ in case 2, resulting in a reduced SWA tracking error compared to case 1 when close to the zero angular velocity.

**Figure 7:**
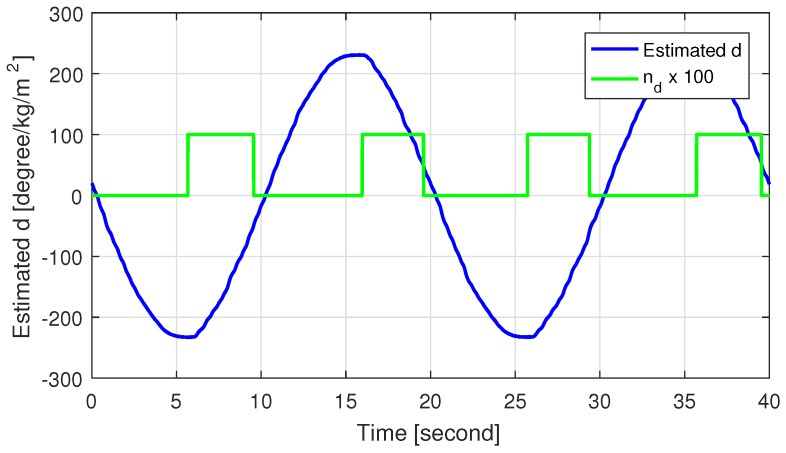
Estimated self-aligning torque in case 2.

**Figure 8:**
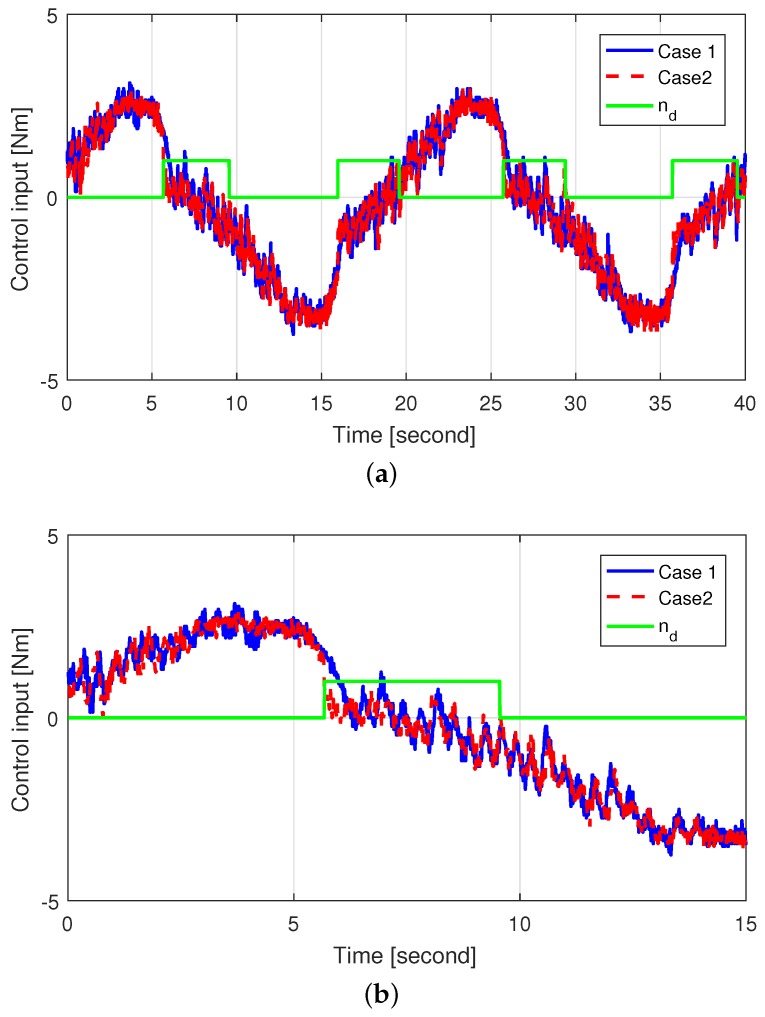
Control input in cases 1 and 2 (**a**) Zoomed-out and (**b**) Zoomed-in.

Figure 7 shows the self-aligning torque estimated by the HGDOB for case 2, and Figure 8 shows the control inputs for both cases. The amplitude of the self-aligning torque increased when the steering wheel turned outward. In addition, the sign of the self-aligning torque was opposite to that of the derivative of the control input, thus cancelling the self-aligning torque. On the other hand, the self-aligning torque decreased when the steering wheel turned inward, reducing its amplitude. In addition, the sign of the self-aligning torque was the same as that of the derivative of the control input, thus contributing to the control of the SWA. Consequently, the self-aligning torque was activated for damping, and thus the control input for case 2 reduced compared to that for case 1 when the self-aligning torque was applied, especially after reaching the zero angular velocity. Overall, we see both reduction of the control input and improved SWA tracking performance using self-aligning torque in case 2 by applying the proposed control method.

## 6. Conclusions

We propose a nonlinear SWA control method using the self-aligning torque for EPS in lateral control systems of autonomous vehicles. We designed an HGDOB to estimate the self-aligning torque without relying on the measured signal derivative. The nonlinear controller was designed by backstepping to bound the SWA tracking error. In the proposed controller, the self-aligning torque induced damping to improve the tracking performance of the controller when the input torque had the same direction of the self-aligning torque. Experimental results validated the performance of the proposed control method via an EPS hardware-in-the-loop simulation system. Specifically, we verified the improvement in SWA tracking performance by using the self-aligning torque in the proposed controller. Moreover, the SWA tracking error reduced given the damping provided by the self-aligning torque when the SWA was returning to the center position. Furthermore, the control input reduced during this period by the contribution of the self-aligning torque in returning the SWA to the center position.

## Nomenclature

$\theta_{h}$	Steering wheel angular position [deg]
$\theta_{h_{d}}$	Desired steering wheel angular position [deg]
$\omega_{h}$	Steering wheel angular velocity [deg/s]
$\theta_{m}$	Motor angular position [deg]
$\omega_{m}$	Motor angular velocity [deg/s]
*i*	Current input of the motor [A]
*T*	Input torque of electric power steering system ($T=K_{t}i$) [N·m]
$K_{t}$	Motor torque constant [N·m/A]
$T_{d}$	Driver torque [N·m]
$T_{s}$	Self-aligning torque [N·m]
$J_{c}$	Steering column moment of inertia [Kg·m^2^]
$B_{c}$	Steering column viscous damping [N·m/(rad/s)]
$K_{c}$	Steering column stiffness [N·m/rad]
$M_{r}$	Mass of the rack [kg]
$B_{r}$	Viscous damping of the rack [N·m/(rad/s)]
$R_{p}$	Steering column pinion radius [m]
$K_{r}$	Tire spring rate [N/m]
$J_{m}$	Motor moment of inertia [kg·m^2^]
$B_{m}$	Motor shaft viscous damping [N·m/(rad/s)]
$V_{x}$	Longitudinal velocity [Km/h]
